# Magnetic Filler Polymer Composites—Morphology Characterization and Experimental and Stochastic Finite Element Analyses of Mechanical Properties

**DOI:** 10.3390/polym15132897

**Published:** 2023-06-30

**Authors:** Yingnan Wang, Hamidreza Ahmadi Moghaddam, Jorge Palacios Moreno, Pierre Mertiny

**Affiliations:** Department of Mechanical Engineering, University of Alberta, Edmonton, AB T6G 1H9, Canada; yingnan1@ualberta.ca (Y.W.); ahmadimo@ualberta.ca (H.A.M.); ajorge@ualberta.ca (J.P.M.)

**Keywords:** magnetic filler polymer composites, NdFeB particles, epoxy polymer, morphology, mechanical properties, tensile testing, numerical modeling, stochastic finite element analysis

## Abstract

Polymer composites containing magnetic fillers are promising materials for a variety of applications, such as in energy storage and medical fields. To facilitate the engineering design of respective components, a comprehensive understanding of the mechanical behavior of such inhomogeneous and potentially highly anisotropic materials is important. Therefore, the authors created magnetic composites by compression molding. The epoxy polymer matrix was modified with a commercial-grade thickening agent. Isotropic magnetic particles were added as the functional filler. The microstructural morphology, especially the filler distribution, dispersion, and alignment, was characterized using microscopy techniques. The mechanical properties of the composites were experimentally characterized and studied by stochastic finite element analysis (SFEA). Modeling was conducted employing four cases to predict the elastic modulus: fully random distribution, randomly aligned distribution, a so-called “rough” interface contact, and a bonded interface contact. Results from experiments and SFEA modeling were compared and discussed.

## 1. Introduction

Polymer composites have widely been used due to their attractive properties, including low density and good mechanical properties [1]. Incorporating additives can improve the rheological behavior of the polymer matrix, which can expand opportunities in terms of designing and fabricating the polymer composite. Functional composites can be composed with a variety of different fillers. For instance, an electrically conducting polymer polypyrrole can be combined with other biopolymers/nanomaterials by solvent casting, in situ polymerization, and electrospinning to improve physicochemical, mechanical, and biological properties [2]; and graphene nanoplatelets can be used to tailor epoxy composites to have improved thermal, electrical, and mechanical properties for conductive and insulative applications [3].

Magnetic polymer composites are an intensely researched class of materials due to their promise in advancing applications in energy storage [4,5,6,7,8], aerospace [9], and medicine [10]. Additive manufacturing is one of the most popular fabrication methods for magnetic composites. Ling et al. [11] demonstrated a big area additive manufacturing (AM) method to fabricate isotropic neodymium iron boron (NdFeB) powder (65 vol%) reinforced polyamide (35 vol%). Similarly, M. Parans et al. [12] studied the fabrication of polyphenylene sulfide filled with 63 vol% of isotropic NdFeB magnet powders by a big area AM method to enhance thermal, mechanical, and magnetic properties. Kinjal et al. [13] investigated a novel method of an extrusion-based 3D printing AM approach for bonded magnets comprising 65 vol% anisotropic composite powders (NdFeB and samarium–iron–nitrogen (SmFeN)) in polyamide for enhanced magnetic properties. Other scientists, e.g., Pigliaru et al. [14], fabricated NdFeB-loaded polyether ether ketone for both filaments (single-screw extruder) and parts (fused filament fabrication) to investigate the influence of different filler content on the thermal, mechanical, and magnetic properties of printed filaments and parts. Balakrishnan et al. [15] included an additive in epoxy to control magnetic particle settling. The developed formulation was used for in situ polymerization and material jetting-based AM processes. Recently, Ester et al. [16] studied the fabrication of magnetic components based on strontium ferrite and NdFeB (over 90 wt% fillers) by extrusion of magnetic polymer composite materials for permanent magnet applications.

Compared to AM, compression molding is a more accessible method for fabricating magnetic filler-loaded polymer composites. Additionally, compression molding has the potential to achieve higher magnetic density and mechanical properties by applying pressure. In this context, Balakrishnan et al. [17] proposed a methodology to combine magnetic field-induced particle alignment along with a dual-cure resin to create anisotropic magnetic composites through polymer casting and AM. Kaustubh et al. [18] studied the compression molding process for anisotropic NdFeB magnets in a polycarbonate matrix. The fabricated NdFeB polycarbonate composite magnets were shown to have improved mechanical properties. In contemporary studies, the characterization of magnetic composites typically focuses on material morphology and mechanical and magnetic properties, using experimental, analytical, and numerical methods, e.g., finite element analysis (FEA). Notably, in the context of the latter, some of the present co-authors developed a stochastic finite element analysis (SFEA) framework capable of predicting the mechanical, thermal, and electrical properties of particulate-modified polymer composites [19,20,21].

The present study is part of a comprehensive research project that strives to develop magnetic polymer-based composites for electro-mechanical components in kinetic energy storage devices. A thorough understanding of the material mechanical behavior is important to facilitate the engineering design of components made of such multifunctional materials. Consequently, in the present work, an epoxy polymer was modified with magnetic particles and a thickening agent to control particle settling at designated mass fractions. A compression molding method was developed to fabricate isotropic NdFeB magnetic particle-loaded epoxy composites. Samples were tested for mechanical properties by cyclic and tensile tests using a universal testing machine. Numerical modeling was performed employing SFEA to study the material characteristics of the magnetic particle composites. Adopting the advanced modeling approach is considered a novel and promising contribution with the aim of facilitating the engineering design of such inhomogeneous and potentially highly anisotropic materials. In addition to studying mechanical properties, scanning electron microscopy (SEM) and X-ray microscopy were employed to observe the distribution, dispersion, and alignment of magnetic particles within the polymer matrix. Findings from this study are expected to guide the further development of compression molding fabrication methods for high-volume fraction magnetic composites.

## 2. Materials and Methods

### 2.1. Materials

The polymer composite in the present work is composed of isotropic powder of magnetic particles embedded in a thermoset polymer matrix, i.e., an EPON 826/EPICURE W resin system (Miller-Stephenson, Danbury, CT, USA) with 2 wt% of the commercial-grade modifier AEROSIL R202 (Evonik, Parsippany, NJ, USA). EPON 826 is a diglycidyl ether of bisphenol-A epoxy resin with an epoxide equivalent weight of 178–186 while EPIKURE W is methylene dianiline curative with nitrogen content between 15.7% and 15.9%. The epoxy resin and hardener were mixed at a ratio of 100:26.4 by weight. The AEROSIL R202 modifier, a fumed silica surface treated with polydimethysiloxane, was added as an anti-settling agent for the magnetic particles. Isotropic NdFeB magnetic particles (type MQP-15-7, Magnequench, Pendleton, IN, USA) were used as the filler phase. The volume ratio of the magnetic particles and the liquid phase is 12:88. The material density and mass fraction for the various constituent materials are listed in Table 1.

### 2.2. Material Fabrication

Material portions were weighed to a precision of 1 mg for a total batch mass of 100 g. Epoxy resin and hardener were mixed by a SpeedMixer (FlackTek SpeedMixer, Landrum, SC, USA). Then, 2 wt% of AEROSIL R202 additive was mixed into the liquid resin-hardener system, followed by incorporating 12 vol% of magnetic particles into the liquid phase.

Mixing at each step occurred at a speed of 1200 rounds per minute for 2 min. The mixture was transferred into a round steel mold with a diameter of 100 mm. The mold with the mixture was placed inside an oven (Isotemp Oven, Fisher Scientific, Ottawa, ON, Canada) applying heat at 100 °C for 4 h. Heating for an additional 1 hour occurred adding a deadweight with a mass of 4.65 kg to compress the mixture. Then, the mold was temporally removed from the oven and a pressure of 5 tons was applied via a hydraulic press (10-Ton Shop Press by MAXIMUM Canada, Toronto, ON, Canada) for 5 min, which was followed by heating in the oven for another 20 h, that is until the mixture was cured forming solid circular disk. The oven was switched off and the material was allowed to cool to room temperature. The fabricated magnetic polymer samples are herein given the identifier P-12vol%. To characterize the modulus of the matrix phase, samples without any magnetic filler were produced as well (including 2 wt% AEROSIL R202), which are labeled as L-2wt%. After mixing, the liquid phase was cured in the mold, without applying any compression, inside the oven at 100 °C for 24 h.

### 2.3. Material Characterization

Using SEM and X-ray microscopy, the fabricated samples were evaluated for their microstructure, i.e., filler distribution, alignment, and volume fraction. The elastic modulus of samples was measured using cyclic and tensile testing. Specimens with the desired dimensions for the various tests were produced from the circular disks via waterjet cutting (type 2652, OMAX, Kent, WA, USA). To prepare samples for microscopy, an automatic polisher (MultiPrep Polisher, Allied High Tech Products, Cerritos, CA, USA) was used to polish cut surfaces, employing 600 and 1200 grit silicon carbide abrasive pads.

The material stress–strain response was measured according to the standard ASTM D 638-14 [22] using type V dogbone specimens and a universal testing machine (model 5966, Instron, Norwood, MA, USA). Cyclic testing preceded tensile testing. Ten fully reversed tension/compression cycles with a peak load of ±100 N were completed at a speed of 100 N/min. Then, monotonic tensile testing to failure was performed at a speed of 0.25 mm/min. An optical extensometer (type ONE-78PT-200, Epsilon Technology, Jackson, WY, USA) was used to measure strain during cyclic and tensile tests. The elastic modulus was assessed for cyclic and tensile tests based on the stress–strain curves.

The distribution and alignment of magnetic particles inside the composites were probed using field emission SEM at an acceleration voltage of 25 kV (300 VP, Zeiss Sigma, Oberkochen, Germany). The polished sample surface was first coated with carbon, accomplished by a Leica EM SCD005 evaporative carbon coater (Wetzlar, Germany). Digital SEM images were subsequently analyzed. The plugins “Segmentation” and “PAT-GEOM” of the image analysis software ImageJ (National Institutes of Health, Bethesda, MD, USA) were used to identify the particles and analyze particle alignment, respectively.

The arrangement of magnetic particles inside the composite was observed in three dimensions using spatial X-ray microscopy scans (Xradia 620 Versa, Zeiss, Oberkochen, Germany). From scan data, the particle volume fraction was also analyzed using the Dragonfly software (Object Research Systems, Montreal, QB, Canada).

### 2.4. Numerical Modeling

**Figure 1:**
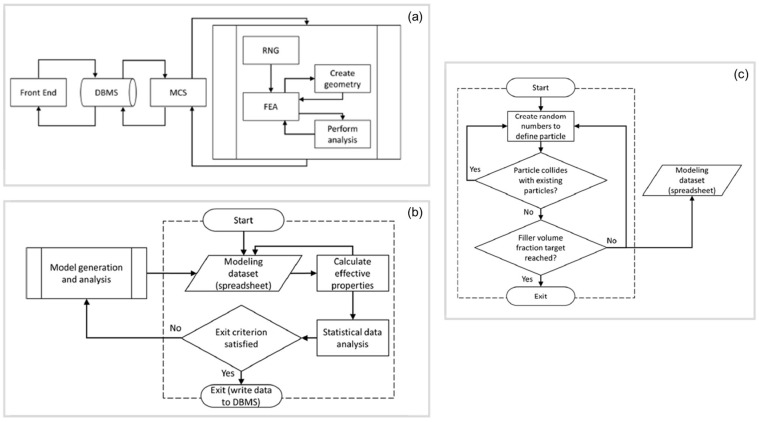
(**a**) Algorithm for the SFEA framework, (**b**) schematic representing the algorithm of Monte Carlo simulation (MCS) module, and (**c**) schematic illustrating the random number generator (RNG) module [19].

In the present work, the SFEA framework described in [19] was adopted to study the mechanical properties of the particulate composite material. In the following, only a synopsis of the SFEA method is provided, and the interested reader is referred to the aforementioned technical literature for further details. The SFEA framework involves a Monte Carlo simulation (MCS) technique to compute results using statistical analysis and different program modules involving multiple programming languages. The SFEA algorithm is shown in Figure 1.

**Figure 2:**
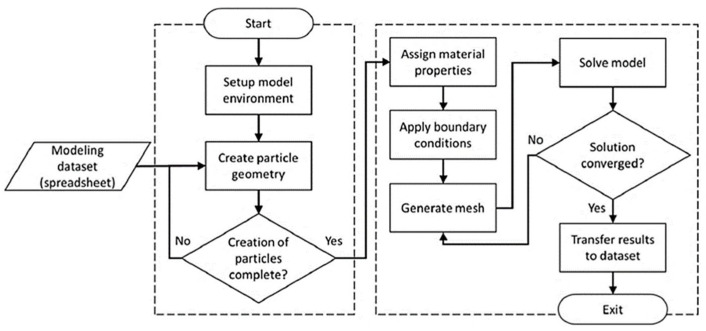
Schematic of the FEA platform for model generation and analysis sub-processes [19].

The transfer of information among the modules used in the modeling framework was developed by applying Visual Basic for Applications programming language. The modeling framework displayed in Figure 1 is explained as follows:

1. The “Front End” module is designed to capture all the input parameters (i.e., size of the representative volume element (RVE), filler and matrix properties, filler particle distribution, etc.), and store them in a database management system (DBMS).
2. The DBMS is developed by an Open Database Connectivity method to ensure the independence of the database for providing accurate information to the MCS module.
3. The MCS module enables storing the information of interest obtained by the analysis sub-process and the model generation. The algorithm in this module (see Figure 1b) is repeated until reaching desired values that satisfy the acceptance criteria (i.e., standard deviation). Once the MCS process stops, the desired property is calculated. Results are calculated in each iteration, and mean values are saved to the database.
4. The random number generator module (RNG), shown in Figure 1c, is created using the programming environment MATLAB (MathWorks, Natick, MA, USA), which facilitates the generation of random numbers required for input variables. These variables are Cartesian coordinates for particle locations (X, Y, Z), and particle dimension and orientation, which enables a random distribution within the RVE. The RNG module also performs collision detection among the particles inside the RVE.
5. The FEA platform is developed using the commercial FEA software ANSYS Workbench (Version 2022R2, ANSYS, Canonsburg, PA, USA). As depicted in Figure 2, the FEA platform possesses two sub-modules to create the three-dimensional geometry and the full FEA model. In the first module, ANSYS DesignModeler in conjunction with scripting in JavaScript language is utilized to read the data generated by the RNG module and create geometries for particles and the RVE. The resulting geometric representation is then transferred to the FEA modeling environment. In the second module, the FEA environment is generated using ANSYS Mechanical in conjunction with JavaScript programming language. In this FEA environment, a convergence study is performed by refining the mesh size and extracting results to check if the convergence is satisfactory. Finally, the results obtained are transferred to the MCS module and saved for further statistical analysis.

The developed SFEA framework was herein adopted to predict the mechanical properties of the magnetic composite considering randomly and aligned distributed flake particles under static-structural conditions. Specifically, flake particles represented in the shape of disks were dispersed in the matrix of modified epoxy with 12 vol%. Properties were tested experimentally or adopted from the technical literature, see details in Table 2.

The particle size distribution was decided based on findings from the microscopy studies. It should be noted that large variations in particle dimensions can drastically increase the computational effort. So, the ranges of particle dimensions were limited to be comparatively narrow, resulting in acceptable times for completing the numerical modeling. It was assumed that all particles fall within a diameter range from 122.2 μm to 135.0 μm. Values for the disk diameters were randomly selected from within this range. For the particle thickness, a data binning approach was applied with four bins to simulate the thickness distribution. For the orientation of particles, two cases were considered: fully random oriented, and fully random aligned. In the latter case, the angle of particles was ±20° with reference to the specimen plane, i.e., the global X and Z direction. The size of the cubical RVE was set at 1100 μm based on a preliminary size effect study for maximizing the randomness of the numerical model [19].

Three-dimensional 10-node (SOLID187) and 20-node (SOLID186) solid structural elements were used for meshing the matrix and particles, respectively. The interface between the particles and matrix was defined by employing three-dimensional 8-node surface-to-surface contact elements (CONTA174 and TARGE170) [19]. In the present study, particle–matrix interfacial contact was assumed considering two distinct cases: “rough contact” and “bonded contact”. For rough contact, separation is permitted but sliding is not. For bonded contact, no relative displacement at the interface is allowed. The following boundary conditions were applied to the RVE based on a mutual Cartesian coordinate system to generate strain and stress in the model. First, a zero-displacement boundary condition was applied at one of the corners located on the RVE back surface, preventing this node from moving along the X, Y, and Z directions (i.e., rigid body motion constraint). Second, the RVE back surface was assigned a displacement boundary condition preventing all nodes located on this surface from moving along the X direction, yet permitting transverse displacement along the Y and Z directions. Third, a displacement by 1 μm along the X direction and free movement in the Y and Z directions was imposed on the nodes on the RVE front surface, thus applying strain to the RVE.

## 3. Results and Discussion

### 3.1. Composite Microstructure Characterization

**Figure 3:**
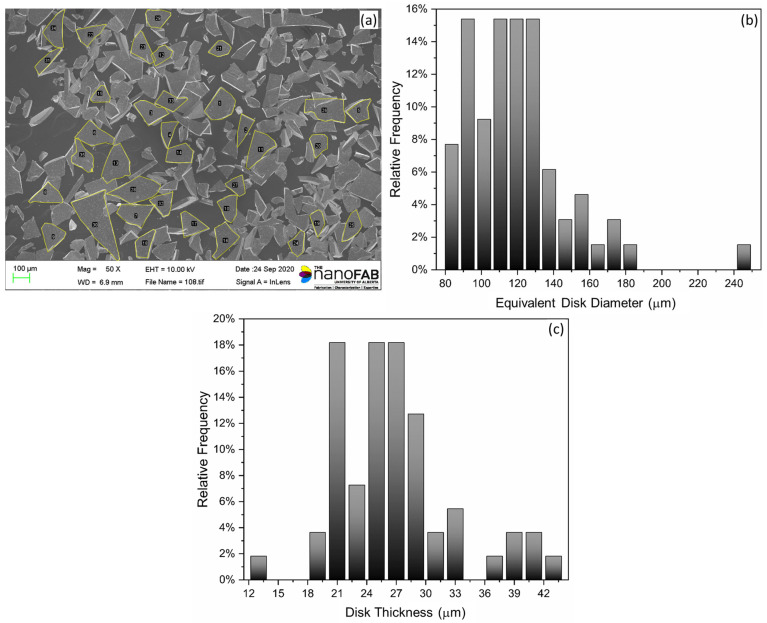
Reduced data from SEM imaging magnetic particle morphology: (**a**) SEM image of magnetic particles, (**b**) histogram of equivalent disk diameters, and (**c**) histogram of disk thickness.

SEM was first performed to study the morphology of the magnetic particles (i.e., dimensional information). Referring to Figure 3, image analysis was performed to determine the filler particle area size and thickness, from which equivalent dimensions were derived to serve as input parameters for numerical modeling. As can be observed in Figure 3a, the particles are shaped irregularly. Table 3 summarizes the results from the filler particle area size and thickness analysis. Comparable dimensions for similar material systems were reported in [24,25,26].

**Figure 4:**
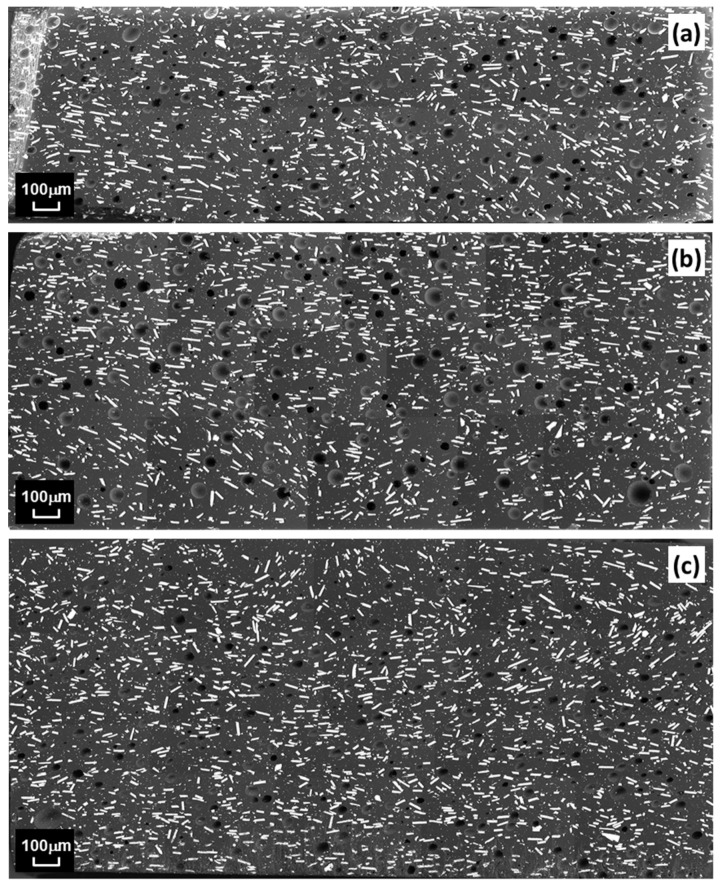
Stitched SEM images showing composite morphology in cut and polished sample surfaces in through-thickness direction, for three specimens, i.e., (**a**) P-12vol%-4, (**b**) P-12vol%-7, and (**c**) P-12vol%-9.

SEM was further used to observe the magnetic particles on the polished surfaces of composite samples in the through-thickness direction of the originally fabricated disks. Figure 4 depicts stitched SEM images elucidating particle distribution, dispersion, and alignment in the matrix. No appreciable settling of magnetic particles due to gravity or application of compression during fabrication could be observed. Additionally, the particles appear to be dispersed well without significant aggregations, i.e., most particles are separated from each other in the continuous phase, and the limited number of existing clusters include only a few particles compared to the overall amount.

**Figure 5:**
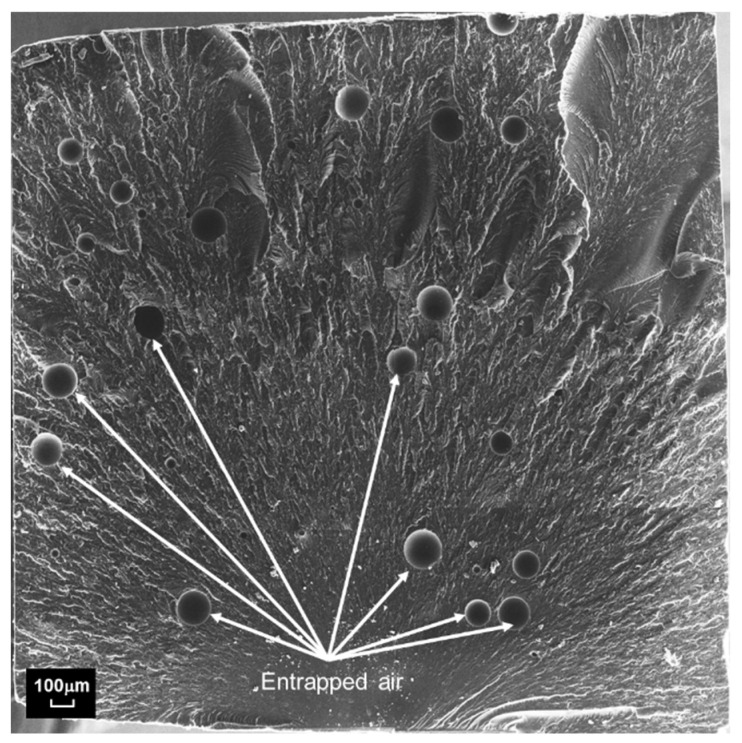
Stitched SEM image showing entrapped air voids in fractured L-2wt% specimen.

Figure 4 exhibits circular features that are interpreted as micro air voids trapped inside the material. Interestingly, entrapped air was also found in L-2wt% samples as shown by the sample fracture surface shown in Figure 5. Note that no vacuum was applied during manufacturing that may have removed entrapped air. Then again, the use of the SpeedMixer device would typically diminish entrained air due to the high acceleration forces that occur when using this mixing technique. It is therefore inferred that the increased viscosity caused by the AEROSIL R202 additive not only prevented particle settling but also raised the susceptibility for entrapped air to remain in the mixture. Note that a vacuum process was considered for fabricating the present samples, however, it was found to be ineffective for the present material system.

**Figure 6:**
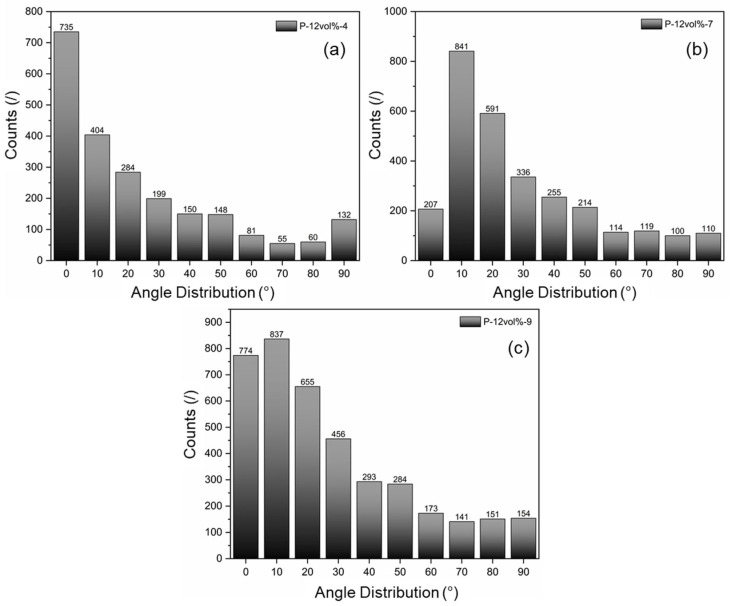
Particle alignment distribution for three samples: (**a**) P-12vol%-4, (**b**) P-12vol%-7, and (**c**) P-12vol%-9.

Upon careful inspection of Figure 4, some level of particle alignment is apparent, supposedly due to the application of compression during the fabrication. Figure 6 depicts the results in terms of alignment angle distribution from image analysis of the SEM images for the three samples shown in Figure 4. The data indicates that the majority of particle orientations falls within the range of 0° to 20° with respect to the in-plane direction. In fact, from the data presented in Table 4, the percentage of particles oriented in the 0° to 20° range is 59.3% (±3.5%). Therefore, the case of randomly aligned particles was included for the numerical analysis, where particle orientation was restricted to fall within the same range.

**Figure 7:**
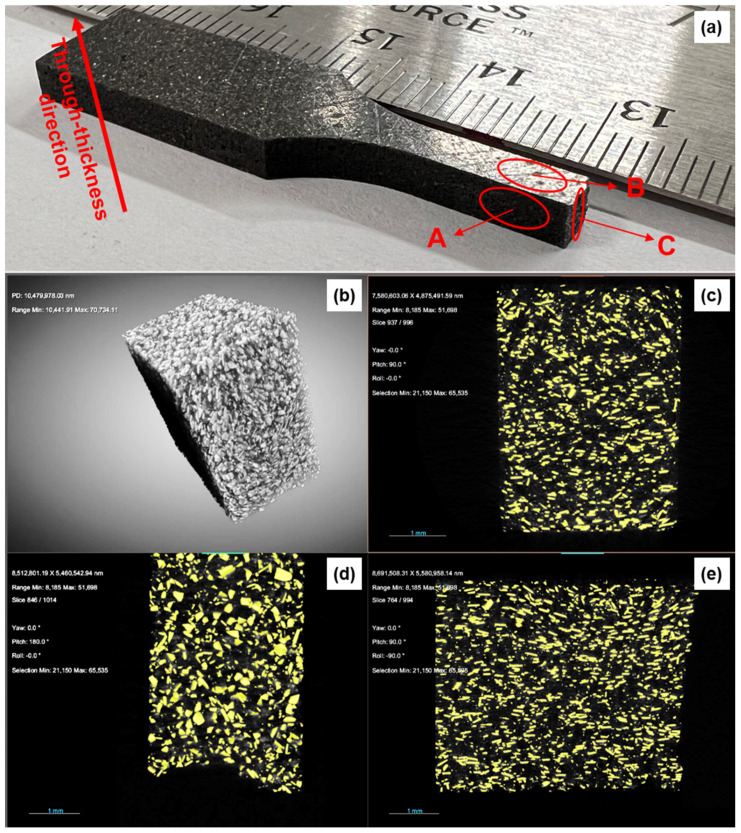
Example images of X-ray microscopy. The photograph of the test sample (**a**) explains the orthogonal cross-section views (**c**–**e**), with (**b**) showing complete sample devoid matrix phase.

Sample morphologies in three-dimensional space were also explored using X-ray microscopy. An example of a scanned sample is shown in Figure 7, including information on image orientations with respect to the sample geometry and orthogonal cross-section views. Notably, the cross-section view for the sample in-plane direction (Figure 7d) exhibits numerous particles in their planar orientation while particles in planar orientation are practically absent in the cross-section views perpendicular to the sample’s in-plane directions (Figure 7c,e), which illustrates the particle alignment effect.

Data from X-ray microscopy were also used to compute the particle volume fraction. For the image analysis, a “seed” was chosen for each particle and then grown to the size of the edge of the particle. The corresponding volume was used for the analysis. Based on this approach, the computed volume fractions were 11.3% and 13.2% for the two samples scanned as part of this study. The average value is therefore 12.3%, which corresponds reasonably well with the nominal sample volume fraction of 12%.

### 3.2. Mechanical Testing

**Figure 8:**
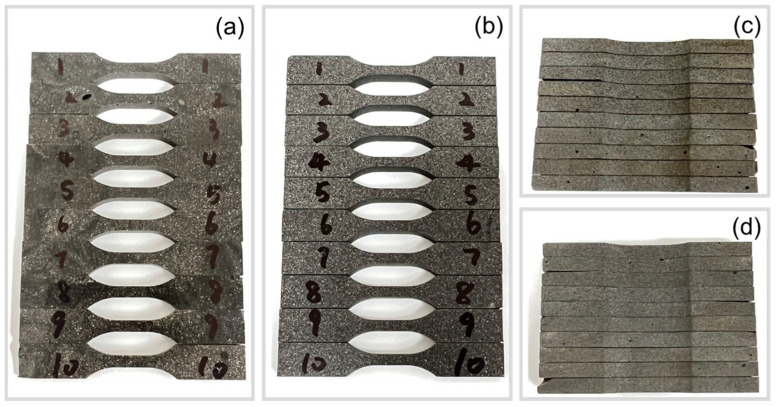
Photographs of tensile test samples: (**a**) top surface, (**b**) bottom surface, (**c**,**d**) side views in through-thickness direction.

Figure 8 shows ten dogbone-shaped samples that were waterjet cut from a fabricated composite disk. While some isolated larger air voids were present in the samples (see Figure 8c,d), the quality of the specimens was deemed sufficiently consistent and suitable for the experiments.

**Figure 9:**
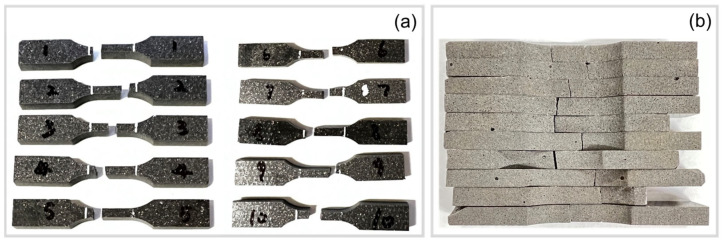
Photographs of test samples after tensile failure: (**a**) top and bottom surface, (**b**) side view.

To study the mechanical properties of the fabricated composites, in particular, the elastic modulus, cyclic and tensile tests were performed using the dogbone specimens. Figure 9 shows the specimens after experiencing tensile failure. It can be observed that failure consistently occurred within the specimen gauge section, which validates the chosen specimen preparation and testing methodology.

**Figure 10:**
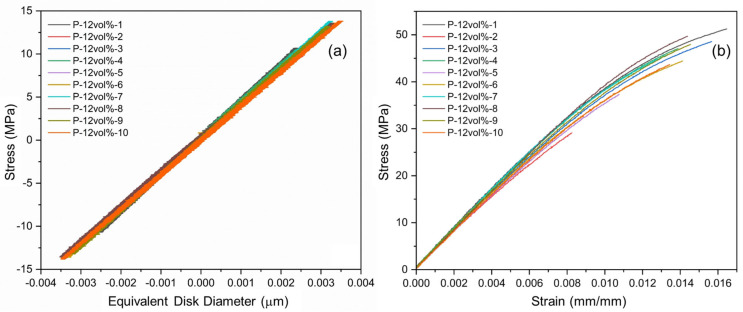
Reduced data from mechanical testing: (**a**) cyclic and (**b**) tensile tests.

**Figure 11:**
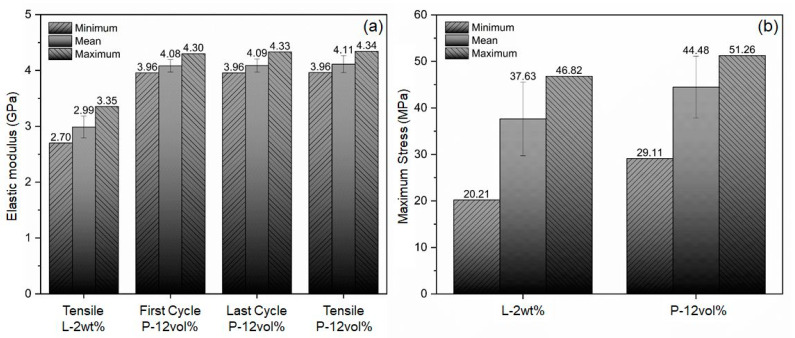
Summary of data from cyclic and tensile mechanical testing: (**a**) elastic modulus, and (**b**) tensile strength. Error bars indicate standard deviations from the mean.

The stress–strain data from cyclic and tensile testing are depicted in the graphs in Figure 10a,b, respectively. Practically no hysteresis effects were observed during the tension/compression cyclic tests, see Figure 10a, indicating that specimen responses were fully elastic, and no appreciable damage accumulated within the selected load range. The slopes of the different stress–strain curves were similar, yet some variation can be observed, which may be the result of slight morphological differences, such as differences in filler alignment and entrapment of air voids. Since material responses were practically linear in the cyclic tests, elastic moduli were computed for the first and last load cycle as the slope of a linear section between the extremes of the applied loading range of ±100 N. As shown in Figure 11a, the calculated average modulus of elasticity for the various composite specimens was 4.08 GPa and 4.09 GPa for the first and last load cycle, with standard deviations of 0.110 GPa and 0.117 GPa, respectively.

In the case of monotonic tensile testing, shown in Figure 10b, elastic moduli were computed for the same tension load range as for the cyclic tests, i.e., 0 to 100 N. An average elastic modulus of 4.11 GPa was obtained for the tensile tests, with a standard deviation of 0.124 GPa, see Figure 11a. For comparison, in [27] a modulus of about 2.0 GPa and 2.5 GPa was reported for the epoxy matrix (without an anti-settling agent) and similar magnetic participle composite system. The reasons for the higher modulus determined in the present study are discussed in the subsequent section on numerical modeling.

While some increase in modulus was observed from cyclic to tensile testing, differences are insignificant (<1%) and are therefore more likely to stem from experimental error rather than damage effects. For comparison, the average elastic modulus for the L-2wt% specimens was 2.99 GPa with a standard variation of 0.195 GPa. As can be expected, the addition of 12 vol% of magnetic filler particles substantially raised the modulus of the composite over the modified polymer system.

In terms of maximum stress reached by the samples in tensile tests, it can be observed that the addition of the magnetic filler resulted in increased values compared to the samples composed solely of the modified polymer system, see Figure 11b. This increase in average maximum stress is quantified as 18.2%. However, significant data variability can be observed for both material systems, indicated by a large difference between minimum and maximum values as well as high standard deviations. These significant variations are likely caused by the rather brittle nature of the resin system combined with detrimental material morphology features, such as entrapped voids and possible filler agglomerations. Therefore, an increase in material strength for the P-12vol% composite over the L-2wt% material cannot be asserted, and further testing and analysis are required to conclusively prove its existence.

### 3.3. Numerical Modeling and Comparison with Experimental Results

**Figure 12:**
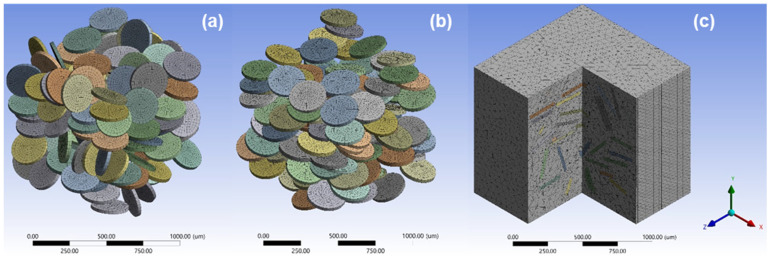
Disk particles inside the RVE for (**a**) fully random distribution and (**b**) randomly aligned distribution cases, with matrix removed, and (**c**) cutaway view of matrix-embedded particles.

For illustration purposes, Figure 12a,b show example images of disk particles for the fully random distribution and randomly aligned distribution cases, respectively. In these images, the matrix was removed from the RVE. Figure 12c depicts a cutaway view revealing matrix-embedded particles inside the RVE.

**Figure 13:**
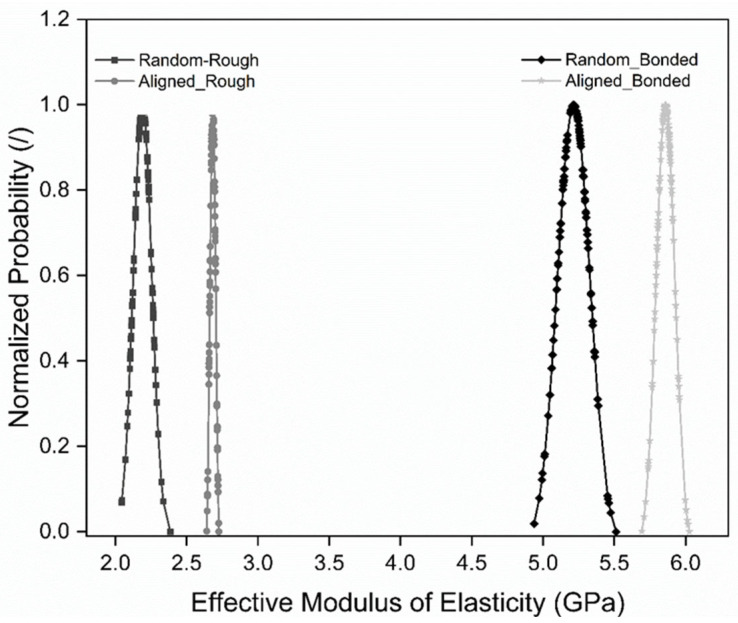
Normalized probability distribution of effective moduli for the four modeling cases, i.e., fully random and randomly aligned distributions with “rough contact” and “bonded contact” options.

Numerical results for the effective elastic modulus from the SFEA are depicted in Figure 13 in terms of normalized probability. It can be noticed that for each modeling case, i.e., the fully random distribution and randomly aligned distribution with the “rough contact” and “bonded contact” options, the modulus data appear to fall under a normal distribution. Table 5 summarized the kurtosis and skewness for the four modeling cases. These values confirm that all modeling data for the elastic modulus are indeed normally distributed [19]. As one may expect, the effective moduli for the randomly aligned distribution cases are greater than for the fully random distribution cases, owing to a greater stiffening effect when the particles are aligned in the loading direction. The difference in interface properties between the rough contact and bonded contact option is shown to have a significant effect on the predicted moduli, i.e., the ability of particles to separate from the matrix in the rough contact cases has a strongly diminishing effect on material stiffness.

**Figure 14:**
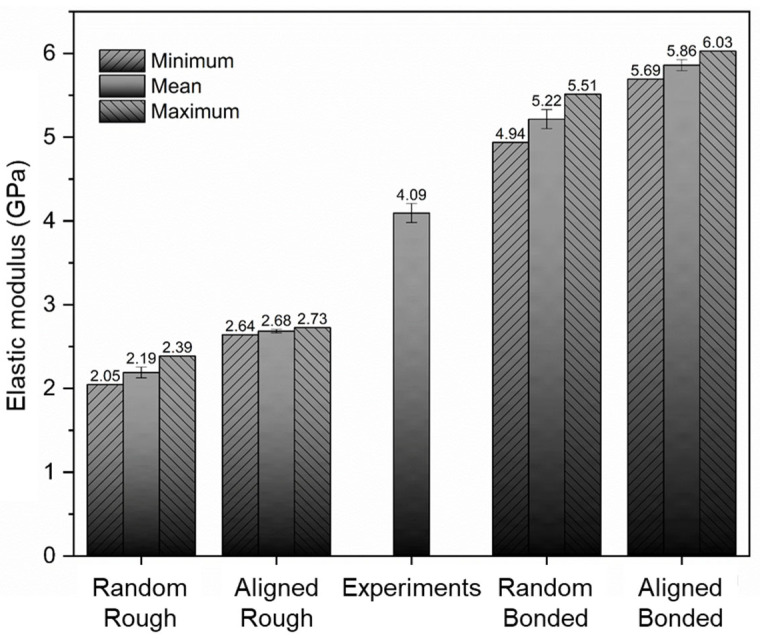
Elasticity moduli from experiments and four numerical modeling cases, i.e., fully random distribution and randomly aligned distribution with “rough contact” and “bonded contact” options.

Figure 14 compares the effective moduli from SFEA modeling with the average value from experiments, that is, 4.09 GPa. Referring again to [27], for a similar material system, an elastic modulus of approximately 2.5 GPa was reported. While a lower modulus of the composite is to be expected due to the absence of the anti-settling agent, it is interesting to note that the reported modulus is close to the numerical results for the fully random distribution and randomly aligned distribution with the “rough contact” interface condition. The authors reported in [27] that particles appeared “attached rather well” to the matrix, yet, the modeling results suggest the absence of a strong chemical bond and substantial mechanical interlocking between filler particles and matrix.

In Figure 14, it can be observed that the experimental average lies between the modeling results for the rough contact and bonded interface contact models. It is hypothesized that in the real composite system, the actual contact condition between the filler particles and the matrix is a “mix” of these two models, i.e., some particles, or parts of the particles, adhere to the matrix, likely due to mechanical interlocking and friction, while other particles separate during loading due to a lack of chemical bonding. Expressing this notion in a quantified manner invoking a simple rule of mixture argument, for the case of the fully random distribution, a mix of particles with 37.3% featuring a rough contact and the remainder having bonded contact would yield a modulus that is equivalent to the experimental results. For the randomly aligned distribution case, the corresponding percentage of particles with rough contact would be 55.7%.

The preceding conjecture neglects that in the actual samples, a high percentage of particles was oriented in the 0° to 20° range, for an average value of *χ* = 59.3% (Table 4). Based on this finding, it is hypothesized that an empirical relation can be derived based on a rule of mixture approach that considers the ratio of aligned particles, *χ*, as well as the ratio of the number of particles with weak matrix interaction (“rough contact”), *α*, and particles with strong matrix interaction (“bonded contact”), 1 − *α*, see Equation (1). Note that this expression neglects any influence that particle directionality may have on particle bonding. Still, the significance of this expression is providing a conservative estimate of the elastic modulus for a given degree of particle alignment.

(1) $$\left[E_{\mathrm{AR}}\alpha +E_{\mathrm{AB}}\left(1-\alpha \right)\right]\chi +\left[E_{\mathrm{RR}}\alpha +E_{\mathrm{RB}}\left(1-\alpha \right)\right]\left(1-\chi \right)=E_{E}$$

where *E*_AR_, *E*_AB_, *E*_RR_ and *E*_RB_ and *E*_E_ are the effective moduli from the modeling cases “aligned rough” (2.68 GPa), “aligned bonded” (5.86 GPa), “random rough” (2.19 GPa) and “random bonded” (5.22 GPa), and the average elastic modulus from experiments (4.09 GPa), respectively (see Figure 14). Simplifying Equation (1), it is found that *α* = 0.484. Based on this expression, the modulus for a composite with fully aligned filler particles (*χ* = 1) and with fully random particles (*χ* = 0) would be correspondingly 4.32 GPa and 3.75 GPa.

The above discussion demonstrates how the SFEA modeling approach while considering distinct composite configurations, is an effective tool to explore material designs and guide development efforts for material fabrication processes. However, it needs to be acknowledged that the presented analysis does not constitute a phenomenological material model. Further research is needed to understand the effect of particle-to-matrix bonding, or lack thereof, and its variation with particle alignment, i.e., whether bonding conditions vary depending on the particle orientation and the exposure of distinct surface areas to the local stress field (particle edges versus planes in platelet-like fillers). Moreover, an idealized particle geometry (circular disk) was employed in the model whereas actual particle shapes were shown to be irregular and ragged. It is currently unknown to what extent the idealized particle geometry affects predictions compared to using a more realistic particle representation. Concessions may also have to be made for artifacts from the fabrication process, namely, entrained air voids.

## 4. Conclusions

In this study, a polymer composite comprising 12% magnetic filler particles by volume and a polymer matrix was successfully fabricated via compression molding. The base epoxy system forming the matrix was modified with an additive to mitigate the settling of the magnetic particles. Microscopic analyses revealed a microstructure of the composite that featured a high degree of particle alignment in the compression plane, i.e., on average 59.3% of particles were aligned within ±20° to the plane. Particles were found to be well-distributed and dispersed in the matrix. Some micro air voids were also observed to exist in the materials, which are believed to have been entrained during the fabrication process.

Samples of both the cured matrix material and the composites were subjected to mechanical testing to assess the modulus of elasticity, yielding mean values of 2.99 GPa and 4.09 GPa, respectively. The average maximum stresses that the matrix material and the composite could sustain were determined as correspondingly 37.6 MPa and 44.5 MPa. It was thus shown that the addition of the magnetic filler particles significantly improved the elastic modulus while avoiding any reduction in mechanical strength. For the latter, it should be mentioned that significant variability in strength was observed among both the matrix material and composite samples, which is ascribed to the brittle nature of the material systems and imperfections such as the entrained air voids and possible minor particle agglomerations.

Numerical analysis was performed employing a stochastic finite element analysis framework based on a Monte Carlo modeling scheme. Four modeling cases were considered for predicting the effective modulus of the composite system: random and aligned filler distributions with either a bonded interface contact or a “rough” contact that permits particle–matrix separation but not interfacial sliding. Predictions using the bonded interface contact were found to exceed the average modulus from experiments, while the “rough” contact configuration underpredicted the test data. An empirical approach based on a rule of mixture concept was proposed that uses the different cases of model predictions to estimate the composite elastic modulus based on the degree of particle alignment and a ratio of bonded versus rough interface contact. While this approach does not claim to constitute a phenomenological material model, the employed analysis facilitates exploring the material system and thus material design and development of fabrication processes.

## Figures and Tables

**Table 1:** Constituent material density and mass fraction for the magnetic composite.

Material	MQP-15-7	EPON 826	EPICURE W	AEROSIL R202
Density (g/cm^3^)	7.61	1.16	1.20	0.60
Mass fraction (%)	47.51	40.70	10.74	1.05

**Table 2:** Matrix and filler material properties used for numerical modeling.

Property	Magnetic Particles	Matrix L-2wt%
Modulus of elasticity (GPa)	160 [23]	2.99 ± 0.20
Particle thickness (μm)	22, 24, 26, 28	/
Particle diameter (μm)	122.2 to 135.0	/

**Table 3:** Reduced data from image analysis for particle equivalent disk diameters and thicknesses.

Property	Particle Equivalent Disk Diameter	Particle Thickness
Average (μm)	128.6	26.2
Standard deviation (μm)	30.3	6.06
Median (μm)	120.4	26.0

**Table 4:** Data from image analysis on particle alignment falling with 0° to 20° orientation with respect to the sample in-plane direction.

Sample ID	Number of Particles in 0°–20° Direction	Total Number of Particles	Fraction of Particles in 0°–20° Direction
P-12vol%-4	1423	2248	63.3%
P-12vol%-7	1639	2887	56.8%
P-12vol%-9	2266	3918	57.8%

**Table 5:** Kurtosis and skewness for the four modeling cases, i.e., fully random distribution and randomly aligned distribution with “rough contact” and “bonded contact” options.

	Fully Random, Rough	Aligned, Rough	Fully Random, Bonded	Aligned, Bonded
Kurtosis	−0.00956	−0.73455	−0.09347	0.2888
Skewness	0.13371	0.07723	0.05985	0.07994

## Data Availability

Data can be made available upon request to the corresponding author.
